# Personalised versus standard text message prompts for increasing trial participant response to telephone follow-up: an embedded randomised controlled retention trial

**DOI:** 10.1186/s13063-024-07916-1

**Published:** 2024-02-07

**Authors:** Esther Herbert, Diana Papaioannou, Amanda Loban, Nikki Totton, Marie Hyslop, Robert Bolt, Christopher Deery

**Affiliations:** 1 Sheffield Centre for Health and Related Research, University of Sheffield, Regent Court, 30 Regent Street, S1 4DA, Sheffield, UK; 2 School of Clinical Dentistry, University of Sheffield, Claremont Crescent, S10 2TA, Sheffield, UK

**Keywords:** Study Within a Trial, Retention, Telephone follow-up, Text messages, Personalised

## Abstract

**Background:**

Improving retention within randomised controlled trials is important. The effectiveness of different strategies can be assessed using a Study Within A Trial (SWAT). Previous research has shown personalised text message reminders improve clinic attendance rates; however, the results are mixed on improving postal questionnaire return. This SWAT aims to assess whether personalised text message reminders improve completion rates for scheduled telephone follow-ups.

**Methods:**

This SWAT is a two-arm, multi-centre randomised controlled trial with equal allocation. The host trial was the Melatonin for Anxiety prior to General anaesthesia In Children trial (ISRCTN 18296119), where the child’s caregiver was to answer a scheduled telephone follow-up 14 days post-surgery; participants for the SWAT were therefore the caregiver. Text messages were sent 24–48 h before the scheduled call and the personalised version contained the first name of the caregiver which was omitted in the non-personalised version. The primary outcome was questionnaire completion rate, defined as the proportion of caregivers successfully contacted, and completed any of the questionnaires, over the telephone within the follow-up window (day 14 + 7 days).

**Results:**

The SWAT included 100 of the 110 (91%) participants randomised into the host trial. Randomisation within the SWAT was equal between non-personalised (*n* = 50) and personalised (*n* = 50) interventions. The overall questionnaire response rate was 73% with a difference between the two interventions of 68% in the non-personalised text message arm and 78% in the personalised text message arm. The adjusted absolute risk difference was 7.1% (95% confidence interval = −10.2%, 24.4%). There was no difference in either the time to response or the number of contact attempts between the two interventions.

**Conclusions:**

There is some evidence that personalised text messages could be effective at increasing response rates when data is collected via telephone and in a population of caregivers for paediatric trial participants. However, similar SWATs have shown mixed results. Given the low-cost and low risks associated with personalising text message reminders, this SWAT could be implemented easily in other RCTs scheduling telephone follow-up appointments.

**Trial Registration:**

ISRCTN 18296119, SWAT 35 (MRC Northern Ireland Network for Trials Methodology Network).

**Supplementary Information:**

The online version contains supplementary material available at 10.1186/s13063-024-07916-1.

## Background

Randomised controlled trials (RCTs) are commonly used in health research to evaluate the effectiveness of treatments. Given the prevalence of the use of RCTs, there is a need to develop and rigorously evaluate strategies for improving retention in these trials to increase efficiency. An assessment of optimum strategies can be completed by embedding a study to test these strategies within a real-life host trial, known as a Study Within a Trial (SWAT) [1].

Potential strategies have been identified specifically to help improve retention in RCTs which includes telephone follow-up instead of postal questionnaires and reminders, via telephone or text message [2]. The host trial already planned to use telephone follow-ups and reminder text messages as it was deemed a simple, cost-effective form of communication that has been shown to improve trial recruitment [3] and return of postal questionnaires in RCTs [4]. Another benefit of text messages is their relative ease of customisability and low cost, allowing personalisation of communication to intended participants.

Personalised text messages, for example using a name or appointment time relevant to the intended recipient, have been shown to increase response in payment of fines [5], re-attendance of high-risk individuals at sexually transmitted infection clinics [6], and monitoring for type 1 diabetes [7]. The effect of personalised text messages specifically on retention in postal questionnaires has been evaluated in other SWATs [8–11] with mixed results. However, it is unknown whether personalised texts are effective where data is collected by other methods and applicable to all populations. This SWAT evaluated the use of personalised text message reminders in a new untested population (caregivers) and the method of data collection (telephone) which have not been previously tested.

The host trial for this SWAT was the MAGIC (Melatonin for Anxiety prior to General anaesthesia In Children) trial (ISRCTN 18296119). The MAGIC trial was a multicentre, parallel-group RCT aimed to evaluate the clinical non-inferiority and cost-effectiveness of melatonin (intervention) against midazolam (usual care) in the premedication of anxious children aged 3 to 14 years prior to general anaesthesia for elective surgeries. The surgical specialities included were dental, ophthalmology, ear-nose-throat, gastroenterology, radiology, plastic, orthopaedic, urology, or other general surgery. As part of the trial, a 14-day follow-up required the child’s caregiver to answer a telephone call and complete three questionnaires over the phone, whilst also providing details on any additional medications prescribed or adverse events that had occurred since their child’s day of surgery. Some additional questionnaires were posted to be self-reported directly by the trial’s older child participants (for example the Child Health Utility—CHU9D) but these were not considered within the results of this SWAT which focussed on the telephone follow-up.

## Methods

### Aim

The aim of this SWAT was to evaluate the effectiveness of a personalised text message including the recipient’s first name versus a standard text message, for prompting response in caregivers to answer and complete the 14-day telephone follow-up questionnaires within the MAGIC trial.

### Design

The SWAT is a two-arm, multi-centre randomised controlled trial with equal allocation (1:1). The SWAT received ethical approval from the Liverpool Central NRES Committee (REC reference 18/NW/0758).

### Participants

Participants were those within the host trial; no separate consent was required to take part in the SWAT. Participants in the host trial were children undergoing elective surgery and the caregivers were also recruited to complete questionnaires around their own anxiety and resource use. Participants for the SWAT were the caregivers only, who answered the telephone follow-up questionnaires on behalf of their child post-surgery.

Inclusion criteria, in addition to that of the host trial, were participants’ caregivers who had access to a mobile phone and provided their number to be contacted by text message and subsequent phone call during their trial participation.

### Intervention

Telephone follow-up calls were scheduled on the day of surgery for 14 days post-surgery. Up to three attempts were made to contact the caregiver until day 21 post-surgery when attempts stopped. Text messages were sent to the participants 24–48 h prior to the scheduled telephone call, for participants randomised to receive a personalised text message (intervention group), the message read:

MAGIC Trial: [First name], you will receive a follow-up phone call in the next 1-2 days for the MAGIC trial. Please be aware that the call may appear from an unknown or withheld number. Your answers are important; so please help by speaking with us. If your child will not be present we ask that they please complete the paper CHU9D form and return it to us in the pre-paid envelope. If you have any questions please call your local trial team. Thanks.

Messages were sent via an internally developed tool, epiSMS, which links our Clinical Database Management System (Prospect) with MessageBird for sending text messages and recording sent details within Prospect.

### Control

As with the intervention group, the control group received a text message, via the same system, 24–48 h prior to the scheduled telephone call. This message, however, was not personalised with the participant’s name; therefore, the message read:

MAGIC Trial: You will receive a follow-up phone call in the next 1-2 days for the MAGIC trial. Please be aware that the call may appear from an unknown or withheld number. Your answers are important; so please help by speaking with us. If your child will not be present we ask that they please complete the paper CHU9D form and return it to us in the pre-paid envelope. If you have any questions please call your local trial team. Thanks

### Outcome measures

The primary outcome is as follows: questionnaire completion rate, defined as the proportion of participants who were successfully contacted and completed any of the questionnaires, over the telephone within the follow-up window (day 14 + 7 days).

Secondary outcomes:

- Time to response, defined as the number of days which elapsed between the reminder text message and the participant being successfully contacted.
- Number of attempts to contact, defined as the number of text messages or telephone calls required before the participant was successfully contacted with completion of at least one questionnaire.

### Randomisation and blinding

Participants were randomly allocated (1:1) to either receive non-personalised text message reminders or personalised text message reminders using a randomisation list pre-generated by a statistician. The trial team randomised the participants to the SWAT following randomisation to the host trial using lists created by the statistician. Randomisation lists were created using block randomisation (block size 2) and stratified by treatment allocation within the host trial.

Participants were blind to the SWAT allocation; however, no blinding of the trial team was present due to the nature of the intervention and logistics of the SWAT.

### Sample size

As is common with SWATs, the sample size was bound by the host trial. Recruitment to the SWAT was expected to be 455 patients (73% of the host trial target) and it was estimated this would provide 80% power to detect differences in completion rates of 8% or more (assuming a control completion of 83%), which was felt to be an important and realistic difference.

### Analysis

Baseline data were summarised by group allocation using appropriate descriptive statistics. Binary logistic regression was used to assess questionnaire completion rates between the two groups, adjusting for host trial treatment allocation and gender. Resulting proportions, percentages, odds ratios, and 95% confidence intervals for the difference between groups are presented.

Time to response was assessed between the groups using Cox regression, adjusted for host trial treatment allocation and gender. Data is presented as a hazard ratio and related 95% confidence intervals. Participants lost to follow-up after their text was sent were censored at that point. The number of attempts to contact is analysed using a negative binomial regression, adjusting for host trial treatment allocation and gender. Resulting mean differences and 95% confidence intervals are calculated.

An intention-to-treat (ITT) population was used for all analyses. It comprised all randomised participants regardless of whether they were sent a text message or withdrew from the host trial prior to their 14-day follow-up. All analyses used two-sided tests and were assessed at the 5% significance level. Analysis was completed within R Version 4.1.0.

## Results

**Fig. 1:**
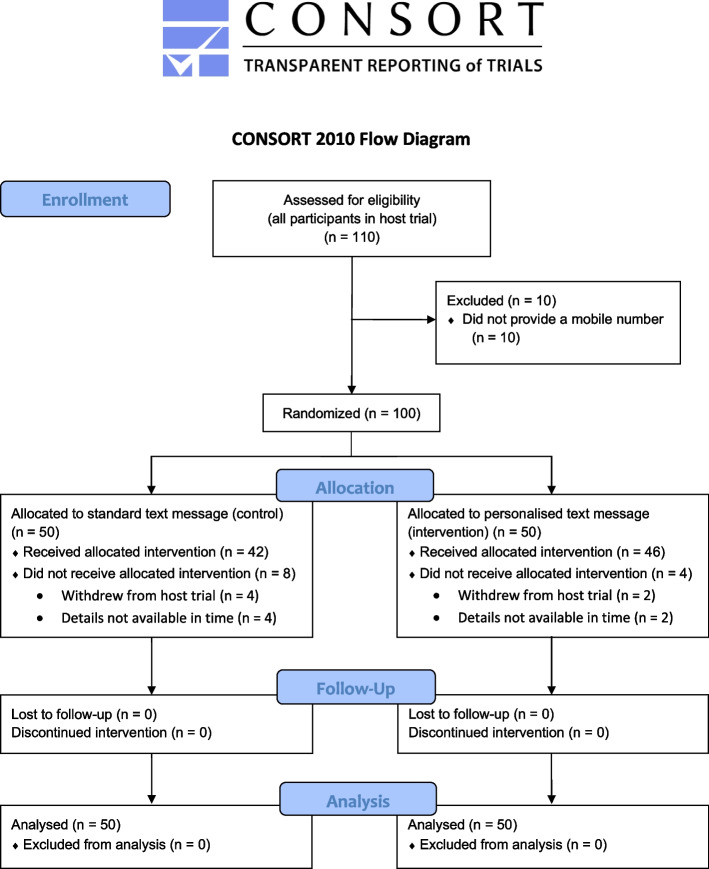
CONSORT flowchart for the SWAT

The MAGIC host trial opened in July 2019 and closed early in November 2022 after 17 months of non-consecutive recruitment. This early closure was due to issues following the COVID-19 pandemic including delays and limitations in identifying and recruiting eligible participants for elective surgery, as well as resourcing constraints regarding the supply of the drug (midazolam). Given this, the SWAT analysis presented was not powered as intended and therefore *p*-values have not been included within the results. The host trial concluded with a total of 110 participants; of these, 100 (91%) were also within the SWAT (50 = standard text message, 50 = personalised text message). Figure 1 shows the flow of participants through the SWAT.

Inclusion to the SWAT differed slightly between the two host trial arms, with 95% and 87% of the control and intervention groups respectively included (see Supplementary material). However, the treatment allocation in the host trial was blinded to both participants and the trial team and consent to the SWAT was not separate from that of the main trial. No reasons were recorded for non-inclusion to the SWAT. Six participants (6%) withdrew from the host trial, and therefore the SWAT, between randomisation and surgery. Overall, 88% of participants received the SWAT intervention (84% in the standard text message arm and 92% in the personalised text message arm). One person was called in error before a text message was sent but remains in the analysis as per the ITT principle.

### Baseline characteristics

The baseline characteristics (Table 1) were similar across both groups with an overall mean (SD) age of 38 (7.8) years, the majority being female (87%) and the biological mother (84%) of the child participating in the host trial.

**Table 1:** Baseline characteristics of the SWAT participants

		Standard text message *N* = 50	Personalised text message *N* = 50	Total *N* = 100
Age (years)	Mean (SD)	37.5 (8.2)	38.5 (7.3)	38.0 (7.8)
	Median (IQR)	36.5 (31.0, 42.0)	38.0 (33.2, 41.8)	37.5 (32.0, 42.0)
Sex	Male	8 (16%)	5 (10%)	13 (13%)
	Female	42 (84%)	45 (90%)	87 (87%)
Relationship to the child	Biological parent (mother)	41 (82%)	43 (86%)	84 (84%)
	Biological parent (father)	7 (14%)	6 (12%)	13 (13%)
	Grandparent	1 (2%)	1 (2%)	2 (2%)
	Adoptive parent	1 (2%)	0 (0%)	1 (1%)

### Questionnaire completion rate

The overall questionnaire completion rate of the 14-day telephone follow-up (within a window of plus 7 days) was 73% across all participants in the SWAT (Table 2). This differed slightly between the two groups with a completion rate of 68% and 78% for the standard vs personalised groups respectively. This analysis resulted in an adjusted absolute difference of 7.1% (95% CI = −10.2 to 24.4%).

**Table 2:** Results of the analysis comparing the treatment groups on the primary and secondary outcomes

		Standard text message *N* = 50	Personalised text message *N* = 50	Total *N* = 100	Results
Questionnaire completed in the follow-up window?	Yes	34 (68%)	39 (78%)	73 (73%)	aOR (95% CI) = 1.49 (0.56, 4.09) aRD (95% CI) = 7.1% (−10.2%, 24.4%)
	No	12 (24%)	9 (19%)	21 (21%)	
	Withdrew prior to follow-up	4 (8%)	2 (4%)	6 (6%)	
Time to response (days)	*n*	32	39	71	aHR (95% CI) = 0.94 (0.58, 1.52)
	Mean (SD)	3.2 (2.7)	3.4 (4.7)	3.3 (3.9)	
	Median (IQR)	2.0 (2.0, 3.0)	2.0 (2.0, 3.0)	2.0 (2.0, 3.0)	
Number of contact attempts	*n*	36	41	77	aIRR (95% CI) = 0.87 (0.6, 1.26)
	Mean (SD)	1.6 (1.0)	1.4 (0.8)	1.5 (0.9)	
	Median (IQR)	1.0 (1.0, 2.0)	1.0 (1.0, 2.0)	1 (1.0, 2.0)	

*aOR* adjusted odds ratio, *aRD* adjusted risk difference, *aHR* adjusted hazard ratio, *aIRR* adjusted incidence rate ratio, *CI* confidence interval, *SD* standard deviation, *IQR* interquartile range

### Time to response and number of contact attempts

For those participants that were successfully contacted, there was no difference between the two groups on either the time to response (aHR 0.94; 95% CI = 0.58 to 1.52) or the number of contact attempts (aIRR 0.87; 95% CI = 0.6 to 1.26).

### Costs

The cost is the same for personalised or non-personalised text messages through the system used in the host trial. However, there was an additional time requirement from the data management and software development team to set up and test the automated process of personalising the text messages with the caregiver’s first name, although this was minimal. Additionally, the process requires the use of potentially identifiable information (in this case limited to first name) in an additional way to usual trial procedures and requirements which should be considered. Despite this, personalising the text messages was relatively straightforward with low-cost implications.

## Discussion

The use of personalised text message reminders could be effective in improving the completion rate of scheduled telephone interview follow-up appointments in an RCT where the population receiving the text messages were caregivers to paediatric trial participants. The better response rate (an adjusted absolute difference of 7.1%) was found in the personalised text message group; however, the confidence interval around this was large. There is no known evidence on what constitutes a meaningful difference in retention rate in RCTs; however, we consider an improvement of 7% to be an important difference. Given the low-cost and low risks associated with personalising text message reminders, this intervention could be implemented easily in RCTs sending text message reminders.

Four SWATs have previously evaluated personalised messaging in relation to questionnaire completion and provide mixed results on its effectiveness. Three of the SWATs related to postal questionnaires; two of these did not find any difference when using personalised messaging [9, 10]. However, one SWAT with postal questionnaires found an 8.1% difference in postal questionnaire response rate [8] which is comparable with the findings from this study. The authors do not provide suggestions as to why the larger 8.1% difference was seen within their SWAT [8], and not the other two postal questionnaire SWATS [9, 10]. The fourth SWAT [11] was the personalisation of text messages prior to a telephone follow-up; findings were in favour of non-personalised text messages (adjusted OR 0.44). However, the study population was very different from our own. The variability in these results reflects the uncertainty in estimates of the effect of the intervention.

There was no difference in the time of contact or number of contact attempts between the two groups. As there was one contact per day, this suggests the participants were responding on the same day in both groups but more likely to respond in the personalised message group.

### Limitations

As the host trial closed early, the sample size was lower than expected and therefore statistically significant results were no longer expected. This has led to large confidence intervals around the primary outcome difference. Further research is required to confirm the findings in this setting.

Data from this SWAT will be added to the PROMETHEUS repository for SWATs to allow meta-analysis to take place with other similar interventions.

## Conclusions

Despite not being statistically significant, there is some evidence that personalised text messages can be effective at increasing response rates when data is collected via telephone and in a population of caregivers for paediatric trial participants. Given the low-cost and low risks associated with personalising text message reminders, this intervention could be implemented easily in RCTs scheduling telephone follow-up appointments.

### Supplementary Information

**Additional file 1:** **Table A.** Recruitment to the SWAT, overall and by host trial treatment group.

## Data Availability

The datasets used and analysed during the current study will be made available from the PROMETHEUS repository for SWATs.

## Abbreviations

aHR: Adjusted hazard ratio
aIRR: Adjusted incidence rate ratio
aOR: Adjusted odds ratio
aRD: Adjusted risk difference
CI: Confidence interval
ISRCTN: International standard randomised controlled trial number
ITT: Intention-to-treat
IQR: Interquartile range
MAGIC: Melatonin for anxiety prior to general anaesthesia in children
PP: Per protocol
RCT: Randomised controlled trial
SD: Standard deviation
SWAT: Study Within a Trial
