# Supplementary material for: Personalised versus standard text message prompts for increasing trial participant response to telephone follow-up: an embedded randomised controlled retention trial
Source: Trials. 2024 Feb 7;25:108. doi: 10.1186/s13063-024-07916-1 (PMC10848401; doi:10.1186/s13063-024-07916-1)
Supplement: Supplementary file 1 — Additional file 1: Table A. Recruitment to the SWAT, overall and by host trial treatment group. [file 13063_2024_7916_MOESM1_ESM.docx]

Table A: Recruitment to the SWAT, overall and by host trial treatment group

|  |  | Host Trial Treatment Group | | Total  N = 110 |
| --- | --- | --- | --- | --- |
|  |  | Control (Midazolam)  N = 55 | Intervention (Melatonin)  N = 55 |  |
| Randomised to SWAT study | Yes | 52 (95%) | 48 (87%) | 100 (91%) |
|  | No | 3 (5%) | 7 (13%) | 10 (9%) |
| SWAT study group | Standard text message | 27 (52%) | 23 (48%) | 50 (50%) |
|  | Personalised text message | 25 (48%) | 25 (52%) | 50 (50%) |
